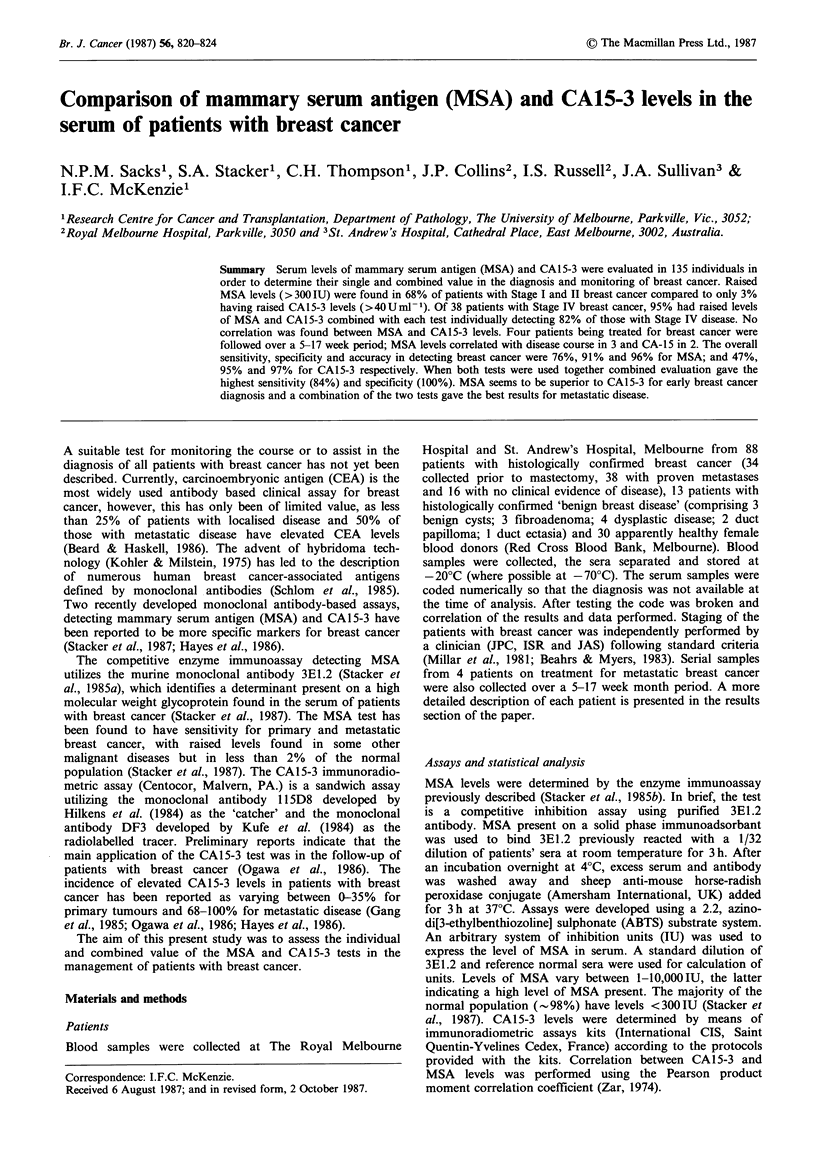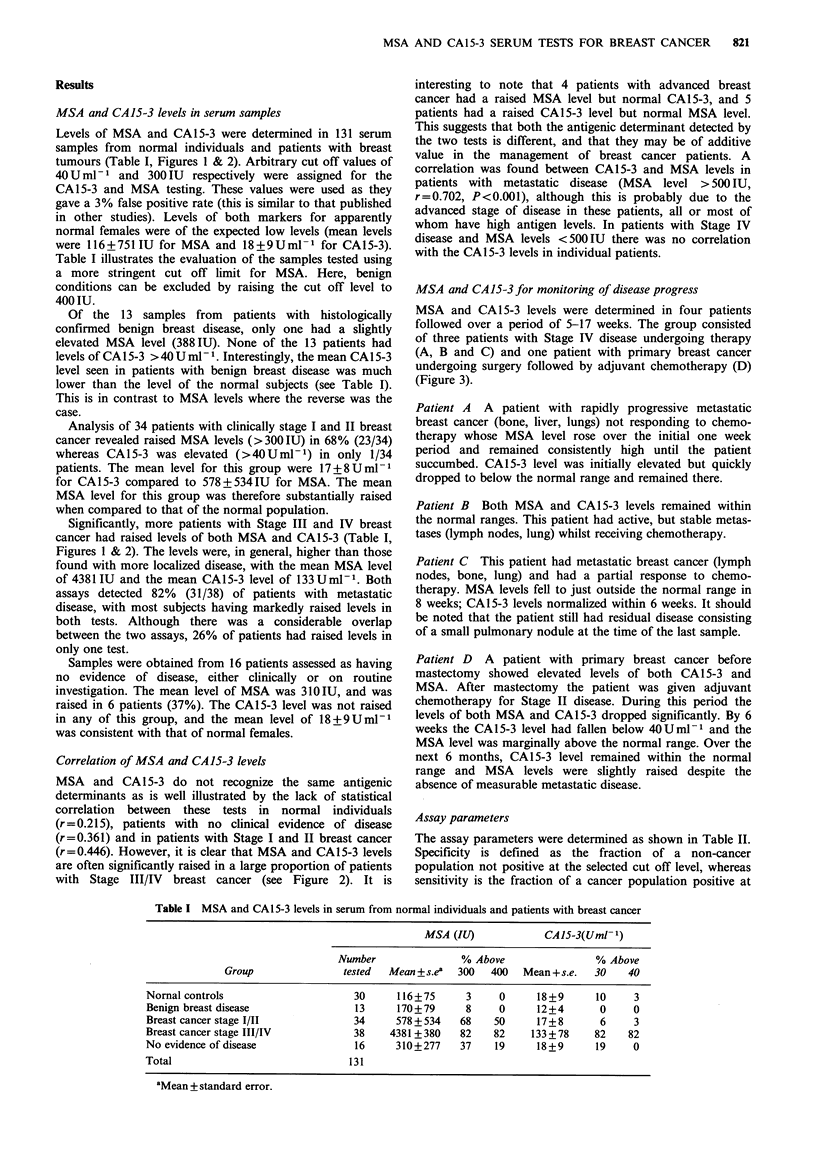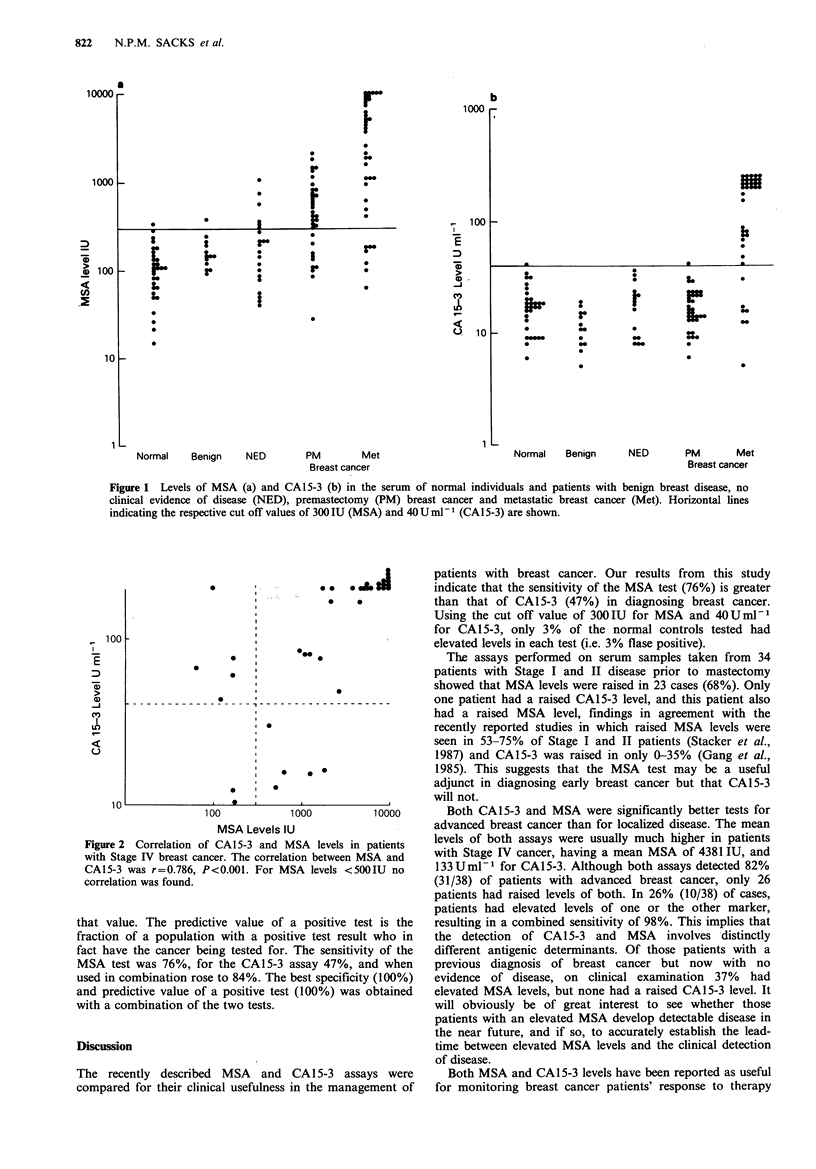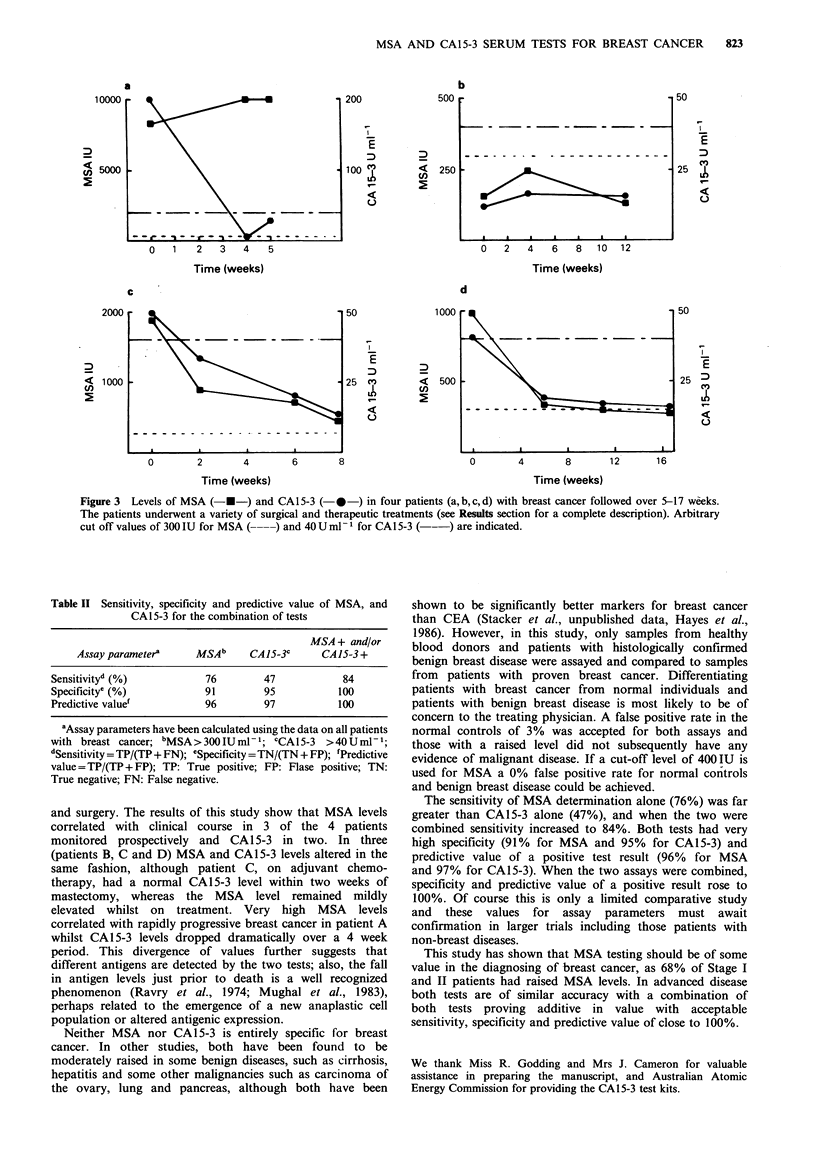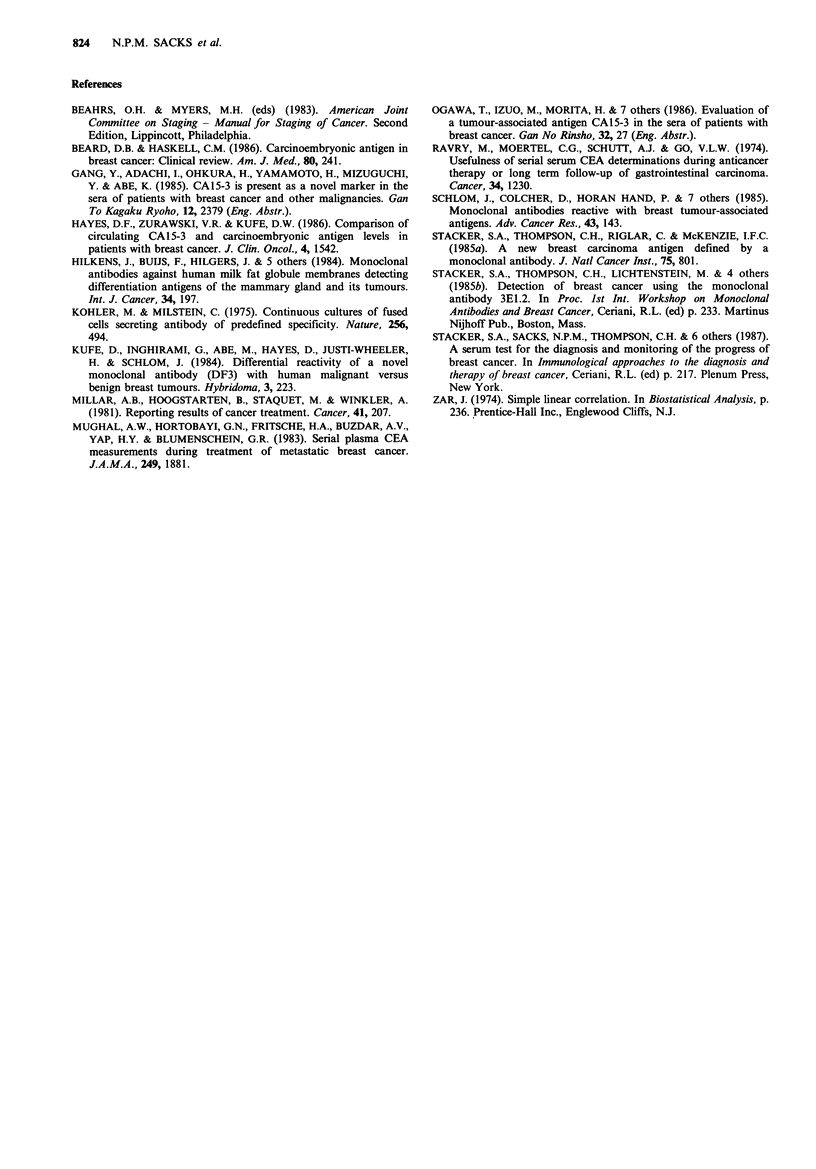# Comparison of mammary serum antigen (MSA) and CA15-3 levels in the serum of patients with breast cancer.

**DOI:** 10.1038/bjc.1987.297

**Published:** 1987-12

**Authors:** N. P. Sacks, S. A. Stacker, C. H. Thompson, J. P. Collins, I. S. Russell, J. A. Sullivan, I. F. McKenzie

**Affiliations:** Department of Pathology, University of Melbourne, Parkville, Vic., Australia.

## Abstract

Serum levels of mammary serum antigen (MSA) and CA15-3 were evaluated in 135 individuals in order to determine their single and combined value in the diagnosis and monitoring of breast cancer. Raised MSA levels (greater than 300 IU) were found in 68% of patients with Stage I and II breast cancer compared to only 3% having raised CA15-3 levels (greater than 40 U ml-1). Of 38 patients with Stage IV breast cancer, 95% had raised levels of MSA and CA15-3 combined with each test individually detecting 82% of those with Stage IV disease. No correlation was found between MSA and CA15-3 levels. Four patients being treated for breast cancer were followed over a 5-17 week period; MSA levels correlated with disease course in 3 and CA-15 in 2. The overall sensitivity, specificity and accuracy in detecting breast cancer were 76%, 91% and 96% for MSA; and 47%, 95% and 97% for CA15-3 respectively. When both tests were used together combined evaluation gave the highest sensitivity (84%) and specificity (100%). MSA seems to be superior to CA15-3 for early breast cancer diagnosis and a combination of the two tests gave the best results for metastatic disease.


Br. J. Cancer (1987) 56, 820–824
© The Macmillan Press Ltd., 1987

### Comparison of mammary serum antigen (MSA) and CA15-3 levels in the serum of patients with breast cancer

N.P.M. Sacks[1], S.A. Stacker[1], C.H. Thompson[1], J.P. Collins[2], I.S. Russell[2], J.A. Sullivan[3] & I.F.C. McKenzie[1]

[1]*Research Centre for Cancer and Transplantation, Department of Pathology, The University of Melbourne, Parkville, Vic., 3052;* [2]*Royal Melbourne Hospital, Parkville, 3050 and* [3]*St. Andrew's Hospital, Cathedral Place, East Melbourne, 3002, Australia.*

**Summary** Serum levels of mammary serum antigen (MSA) and CA15-3 were evaluated in 135 individuals in order to determine their single and combined value in the diagnosis and monitoring of breast cancer. Raised MSA levels (>300 IU) were found in 68% of patients with Stage I and II breast cancer compared to only 3% having raised CA15-3 levels (>40 U ml$^{-1}$). Of 38 patients with Stage IV breast cancer, 95% had raised levels of MSA and CA15-3 combined with each test individually detecting 82% of those with Stage IV disease. No correlation was found between MSA and CA15-3 levels. Four patients being treated for breast cancer were followed over a 5–17 week period; MSA levels correlated with disease course in 3 and CA-15 in 2. The overall sensitivity, specificity and accuracy in detecting breast cancer were 76%, 91% and 96% for MSA; and 47%, 95% and 97% for CA15-3 respectively. When both tests were used together combined evaluation gave the highest sensitivity (84%) and specificity (100%). MSA seems to be superior to CA15-3 for early breast cancer diagnosis and a combination of the two tests gave the best results for metastatic disease.

A suitable test for monitoring the course or to assist in the diagnosis of all patients with breast cancer has not yet been described. Currently, carcinoembryonic antigen (CEA) is the most widely used antibody based clinical assay for breast cancer, however, this has only been of limited value, as less than 25% of patients with localised disease and 50% of those with metastatic disease have elevated CEA levels (Beard & Haskell, 1986). The advent of hybridoma technology (Kohler & Milstein, 1975) has led to the description of numerous human breast cancer-associated antigens defined by monoclonal antibodies (Schlom *et al.*, 1985). Two recently developed monoclonal antibody-based assays, detecting mammary serum antigen (MSA) and CA15-3 have been reported to be more specific markers for breast cancer (Stacker *et al.*, 1987; Hayes *et al.*, 1986).

The competitive enzyme immunoassay detecting MSA utilizes the murine monoclonal antibody 3E1.2 (Stacker *et al.*, 1985*a*), which identifies a determinant present on a high molecular weight glycoprotein found in the serum of patients with breast cancer (Stacker *et al.*, 1987). The MSA test has been found to have sensitivity for primary and metastatic breast cancer, with raised levels found in some other malignant diseases but in less than 2% of the normal population (Stacker *et al.*, 1987). The CA15-3 immunoradiometric assay (Centocor, Malvern, PA.) is a sandwich assay utilizing the monoclonal antibody 115D8 developed by Hilkens *et al.* (1984) as the 'catcher' and the monoclonal antibody DF3 developed by Kufe *et al.* (1984) as the radiolabelled tracer. Preliminary reports indicate that the main application of the CA15-3 test was in the follow-up of patients with breast cancer (Ogawa *et al.*, 1986). The incidence of elevated CA15-3 levels in patients with breast cancer has been reported as varying between 0–35% for primary tumours and 68–100% for metastatic disease (Gang *et al.*, 1985; Ogawa *et al.*, 1986; Hayes *et al.*, 1986).

The aim of this present study was to assess the individual and combined value of the MSA and CA15-3 tests in the management of patients with breast cancer.

### Materials and methods

### *Patients*

Blood samples were collected at The Royal Melbourne Hospital and St. Andrew's Hospital, Melbourne from 88 patients with histologically confirmed breast cancer (34 collected prior to mastectomy, 38 with proven metastases and 16 with no clinical evidence of disease), 13 patients with histologically confirmed 'benign breast disease' (comprising 3 benign cysts; 3 fibroadenoma; 4 dysplastic disease; 2 duct papilloma; 1 duct ectasia) and 30 apparently healthy female blood donors (Red Cross Blood Bank, Melbourne). Blood samples were collected, the sera separated and stored at −20°C (where possible at −70°C). The serum samples were coded numerically so that the diagnosis was not available at the time of analysis. After testing the code was broken and correlation of the results and data performed. Staging of the patients with breast cancer was independently performed by a clinician (JPC, ISR and JAS) following standard criteria (Millar *et al.*, 1981; Beahrs & Myers, 1983). Serial samples from 4 patients on treatment for metastatic breast cancer were also collected over a 5–17 week month period. A more detailed description of each patient is presented in the results section of the paper.

### *Assays and statistical analysis*

MSA levels were determined by the enzyme immunoassay previously described (Stacker *et al.*, 1985*b*). In brief, the test is a competitive inhibition assay using purified 3E1.2 antibody. MSA present on a solid phase immunoadsorbant was used to bind 3E1.2 previously reacted with a 1/32 dilution of patients' sera at room temperature for 3 h. After an incubation overnight at 4°C, excess serum and antibody was washed away and sheep anti-mouse horse-radish peroxidase conjugate (Amersham International, UK) added for 3 h at 37°C. Assays were developed using a 2.2, azino-di[3-ethylbenthiozoline] sulphonate (ABTS) substrate system. An arbitrary system of inhibition units (IU) was used to express the level of MSA in serum. A standard dilution of 3E1.2 and reference normal sera were used for calculation of units. Levels of MSA vary between 1–10,000 IU, the latter indicating a high level of MSA present. The majority of the normal population (~98%) have levels <300 IU (Stacker *et al.*, 1987). CA15-3 levels were determined by means of immunoradiometric assays kits (International CIS, Saint Quentin-Yvelines Cedex, France) according to the protocols provided with the kits. Correlation between CA15-3 and MSA levels was performed using the Pearson product moment correlation coefficient (Zar, 1974).

Correspondence: I.F.C. McKenzie.
Received 6 August 1987; and in revised form, 2 October 1987.

### Results

### *MSA and CA15-3 levels in serum samples*

Levels of MSA and CA15-3 were determined in 131 serum samples from normal individuals and patients with breast tumours (Table I, Figures 1 & 2). Arbitrary cut off values of 40 U $ml^{-1}$ and 300 IU respectively were assigned for the CA15-3 and MSA testing. These values were used as they gave a 3% false positive rate (this is similar to that published in other studies). Levels of both markers for apparently normal females were of the expected low levels (mean levels were 116±751 IU for MSA and 18±9 U $ml^{-1}$ for CA15-3). Table I illustrates the evaluation of the samples tested using a more stringent cut off limit for MSA. Here, benign conditions can be excluded by raising the cut off level to 400 IU.

Of the 13 samples from patients with histologically confirmed benign breast disease, only one had a slightly elevated MSA level (388 IU). None of the 13 patients had levels of CA15-3 >40 U $ml^{-1}$. Interestingly, the mean CA15-3 level seen in patients with benign breast disease was much lower than the level of the normal subjects (see Table I). This is in contrast to MSA levels where the reverse was the case.

Analysis of 34 patients with clinically stage I and II breast cancer revealed raised MSA levels (>300 IU) in 68% (23/34) whereas CA15-3 was elevated (>40 U $ml^{-1}$) in only 1/34 patients. The mean level for this group were 17±8 U $ml^{-1}$ for CA15-3 compared to 578±534 IU for MSA. The mean MSA level for this group was therefore substantially raised when compared to that of the normal population.

Significantly, more patients with Stage III and IV breast cancer had raised levels of both MSA and CA15-3 (Table I, Figures 1 & 2). The levels were, in general, higher than those found with more localized disease, with the mean MSA level of 4381 IU and the mean CA15-3 level of 133 U $ml^{-1}$. Both assays detected 82% (31/38) of patients with metastatic disease, with most subjects having markedly raised levels in both tests. Although there was a considerable overlap between the two assays, 26% of patients had raised levels in only one test.

Samples were obtained from 16 patients assessed as having no evidence of disease, either clinically or on routine investigation. The mean level of MSA was 310 IU, and was raised in 6 patients (37%). The CA15-3 level was not raised in any of this group, and the mean level of 18±9 U $ml^{-1}$ was consistent with that of normal females.

### *Correlation of MSA and CA15-3 levels*

MSA and CA15-3 do not recognize the same antigenic determinants as is well illustrated by the lack of statistical correlation between these tests in normal individuals ($r=0.215$), patients with no clinical evidence of disease ($r=0.361$) and in patients with Stage I and II breast cancer ($r=0.446$). However, it is clear that MSA and CA15-3 levels are often significantly raised in a large proportion of patients with Stage III/IV breast cancer (see Figure 2). It is interesting to note that 4 patients with advanced breast cancer had a raised MSA level but normal CA15-3, and 5 patients had a raised CA15-3 level but normal MSA level. This suggests that both the antigenic determinant detected by the two tests is different, and that they may be of additive value in the management of breast cancer patients. A correlation was found between CA15-3 and MSA levels in patients with metastatic disease (MSA level >500 IU, $r=0.702$, $P<0.001$), although this is probably due to the advanced stage of disease in these patients, all or most of whom have high antigen levels. In patients with Stage IV disease and MSA levels <500 IU there was no correlation with the CA15-3 levels in individual patients.

### *MSA and CA15-3 for monitoring of disease progress*

MSA and CA15-3 levels were determined in four patients followed over a period of 5–17 weeks. The group consisted of three patients with Stage IV disease undergoing therapy (A, B and C) and one patient with primary breast cancer undergoing surgery followed by adjuvant chemotherapy (D) (Figure 3).

*Patient A* A patient with rapidly progressive metastatic breast cancer (bone, liver, lungs) not responding to chemotherapy whose MSA level rose over the initial one week period and remained consistently high until the patient succumbed. CA15-3 level was initially elevated but quickly dropped to below the normal range and remained there.

*Patient B* Both MSA and CA15-3 levels remained within the normal ranges. This patient had active, but stable metastases (lymph nodes, lung) whilst receiving chemotherapy.

*Patient C* This patient had metastatic breast cancer (lymph nodes, bone, lung) and had a partial response to chemotherapy. MSA levels fell to just outside the normal range in 8 weeks; CA15-3 levels normalized within 6 weeks. It should be noted that the patient still had residual disease consisting of a small pulmonary nodule at the time of the last sample.

*Patient D* A patient with primary breast cancer before mastectomy showed elevated levels of both CA15-3 and MSA. After mastectomy the patient was given adjuvant chemotherapy for Stage II disease. During this period the levels of both MSA and CA15-3 dropped significantly. By 6 weeks the CA15-3 level had fallen below 40 U $ml^{-1}$ and the MSA level was marginally above the normal range. Over the next 6 months, CA15-3 level remained within the normal range and MSA levels were slightly raised despite the absence of measurable metastatic disease.

### *Assay parameters*

The assay parameters were determined as shown in Table II. Specificity is defined as the fraction of a non-cancer population not positive at the selected cut off level, whereas sensitivity is the fraction of a cancer population positive at

**Table I** MSA and CA15-3 levels in serum from normal individuals and patients with breast cancer

| | | *MSA (IU)* | | | *CA15-3(U $ml^{-1}$)* | | |
|---|---|---|---|---|---|---|---|
| Group | *Number tested* | *Mean±s.e*[a] | *% Above* 300 | *% Above* 400 | Mean+*s.e.* | *% Above 30* | *% Above 40* |
| Normal controls | 30 | 116±75 | 3 | 0 | 18±9 | 10 | 3 |
| Benign breast disease | 13 | 170±79 | 8 | 0 | 12±4 | 0 | 0 |
| Breast cancer stage I/II | 34 | 578±534 | 68 | 50 | 17±8 | 6 | 3 |
| Breast cancer stage III/IV | 38 | 4381±380 | 82 | 82 | 133±78 | 82 | 82 |
| No evidence of disease | 16 | 310±277 | 37 | 19 | 18±9 | 19 | 0 |
| Total | 131 | | | | | | |

[a]Mean±standard error.

**Figure 1** Levels of MSA (a) and CA15-3 (b) in the serum of normal individuals and patients with benign breast disease, no clinical evidence of disease (NED), premastectomy (PM) breast cancer and metastatic breast cancer (Met). Horizontal lines indicating the respective cut off values of 300 IU (MSA) and 40 U $ml^{-1}$ (CA15-3) are shown.

**Figure 2** Correlation of CA15-3 and MSA levels in patients with Stage IV breast cancer. The correlation between MSA and CA15-3 was $r=0.786$, $P<0.001$. For MSA levels <500 IU no correlation was found.

that value. The predictive value of a positive test is the fraction of a population with a positive test result who in fact have the cancer being tested for. The sensitivity of the MSA test was 76%, for the CA15-3 assay 47%, and when used in combination rose to 84%. The best specificity (100%) and predictive value of a positive test (100%) was obtained with a combination of the two tests.

### Discussion

The recently described MSA and CA15-3 assays were compared for their clinical usefulness in the management of patients with breast cancer. Our results from this study indicate that the sensitivity of the MSA test (76%) is greater than that of CA15-3 (47%) in diagnosing breast cancer. Using the cut off value of 300 IU for MSA and 40 U $ml^{-1}$ for CA15-3, only 3% of the normal controls tested had elevated levels in each test (i.e. 3% flase positive).

The assays performed on serum samples taken from 34 patients with Stage I and II disease prior to mastectomy showed that MSA levels were raised in 23 cases (68%). Only one patient had a raised CA15-3 level, and this patient also had a raised MSA level, findings in agreement with the recently reported studies in which raised MSA levels were seen in 53–75% of Stage I and II patients (Stacker *et al.*, 1987) and CA15-3 was raised in only 0–35% (Gang *et al.*, 1985). This suggests that the MSA test may be a useful adjunct in diagnosing early breast cancer but that CA15-3 will not.

Both CA15-3 and MSA were significantly better tests for advanced breast cancer than for localized disease. The mean levels of both assays were usually much higher in patients with Stage IV cancer, having a mean MSA of 4381 IU, and 133 U $ml^{-1}$ for CA15-3. Although both assays detected 82% (31/38) of patients with advanced breast cancer, only 26 patients had raised levels of both. In 26% (10/38) of cases, patients had elevated levels of one or the other marker, resulting in a combined sensitivity of 98%. This implies that the detection of CA15-3 and MSA involves distinctly different antigenic determinants. Of those patients with a previous diagnosis of breast cancer but now with no evidence of disease, on clinical examination 37% had elevated MSA levels, but none had a raised CA15-3 level. It will obviously be of great interest to see whether those patients with an elevated MSA develop detectable disease in the near future, and if so, to accurately establish the lead-time between elevated MSA levels and the clinical detection of disease.

Both MSA and CA15-3 levels have been reported as useful for monitoring breast cancer patients' response to therapy

**Figure 3** Levels of MSA (—■—) and CA15-3 (—●—) in four patients (a, b, c, d) with breast cancer followed over 5–17 weeks. The patients underwent a variety of surgical and therapeutic treatments (see **Results** section for a complete description). Arbitrary cut off values of 300 IU for MSA (----) and 40 U ml$^{-1}$ for CA15-3 (——) are indicated.

**Table II** Sensitivity, specificity and predictive value of MSA, and CA15-3 for the combination of tests

| *Assay parameter*[a] | *MSA*[b] | *CA15-3*[c] | *MSA+ and/or CA15-3+* |
|---|---|---|---|
| Sensitivity[d] (%) | 76 | 47 | 84 |
| Specificity[e] (%) | 91 | 95 | 100 |
| Predictive value[f] | 96 | 97 | 100 |

[a]Assay parameters have been calculated using the data on all patients with breast cancer; [b]MSA > 300 IU ml$^{-1}$; [c]CA15-3 > 40 U ml$^{-1}$; [d]Sensitivity = TP/(TP + FN); [e]Specificity = TN/(TN + FP); [f]Predictive value = TP/(TP + FP); TP: True positive; FP: Flase positive; TN: True negative; FN: False negative.

and surgery. The results of this study show that MSA levels correlated with clinical course in 3 of the 4 patients monitored prospectively and CA15-3 in two. In three (patients B, C and D) MSA and CA15-3 levels altered in the same fashion, although patient C, on adjuvant chemotherapy, had a normal CA15-3 level within two weeks of mastectomy, whereas the MSA level remained mildly elevated whilst on treatment. Very high MSA levels correlated with rapidly progressive breast cancer in patient A whilst CA15-3 levels dropped dramatically over a 4 week period. This divergence of values further suggests that different antigens are detected by the two tests; also, the fall in antigen levels just prior to death is a well recognized phenomenon (Ravry *et al.*, 1974; Mughal *et al.*, 1983), perhaps related to the emergence of a new anaplastic cell population or altered antigenic expression.

Neither MSA nor CA15-3 is entirely specific for breast cancer. In other studies, both have been found to be moderately raised in some benign diseases, such as cirrhosis, hepatitis and some other malignancies such as carcinoma of the ovary, lung and pancreas, although both have been shown to be significantly better markers for breast cancer than CEA (Stacker *et al.*, unpublished data, Hayes *et al.*, 1986). However, in this study, only samples from healthy blood donors and patients with histologically confirmed benign breast disease were assayed and compared to samples from patients with proven breast cancer. Differentiating patients with breast cancer from normal individuals and patients with benign breast disease is most likely to be of concern to the treating physician. A false positive rate in the normal controls of 3% was accepted for both assays and those with a raised level did not subsequently have any evidence of malignant disease. If a cut-off level of 400 IU is used for MSA a 0% false positive rate for normal controls and benign breast disease could be achieved.

The sensitivity of MSA determination alone (76%) was far greater than CA15-3 alone (47%), and when the two were combined sensitivity increased to 84%. Both tests had very high specificity (91% for MSA and 95% for CA15-3) and predictive value of a positive test result (96% for MSA and 97% for CA15-3). When the two assays were combined, specificity and predictive value of a positive result rose to 100%. Of course this is only a limited comparative study and these values for assay parameters must await confirmation in larger trials including those patients with non-breast diseases.

This study has shown that MSA testing should be of some value in the diagnosing of breast cancer, as 68% of Stage I and II patients had raised MSA levels. In advanced disease both tests are of similar accuracy with a combination of both tests proving additive in value with acceptable sensitivity, specificity and predictive value of close to 100%.

We thank Miss R. Godding and Mrs J. Cameron for valuable assistance in preparing the manuscript, and Australian Atomic Energy Commission for providing the CA15-3 test kits.

### References

BEAHRS, O.H. & MYERS, M.H. (eds) (1983). *American Joint Committee on Staging – Manual for Staging of Cancer*. Second Edition, Lippincott, Philadelphia.

BEARD, D.B. & HASKELL, C.M. (1986). Carcinoembryonic antigen in breast cancer: Clinical review. *Am. J. Med.*, **80**, 241.

GANG, Y., ADACHI, I., OHKURA, H., YAMAMOTO, H., MIZUGUCHI, Y. & ABE, K. (1985). CA15-3 is present as a novel marker in the sera of patients with breast cancer and other malignancies. *Gan To Kagaku Ryoho*, **12**, 2379 (*Eng. Abstr.*).

HAYES, D.F., ZURAWSKI, V.R. & KUFE, D.W. (1986). Comparison of circulating CA15-3 and carcinoembryonic antigen levels in patients with breast cancer. *J. Clin. Oncol.*, **4**, 1542.

HILKENS, J., BUIJS, F., HILGERS, J. & 5 others (1984). Monoclonal antibodies against human milk fat globule membranes detecting differentiation antigens of the mammary gland and its tumours. *Int. J. Cancer*, **34**, 197.

KOHLER, M. & MILSTEIN, C. (1975). Continuous cultures of fused cells secreting antibody of predefined specificity. *Nature*, **256**, 494.

KUFE, D., INGHIRAMI, G., ABE, M., HAYES, D., JUSTI-WHEELER, H. & SCHLOM, J. (1984). Differential reactivity of a novel monoclonal antibody (DF3) with human malignant versus benign breast tumours. *Hybridoma*, **3**, 223.

MILLAR, A.B., HOOGSTARTEN, B., STAQUET, M. & WINKLER, A. (1981). Reporting results of cancer treatment. *Cancer*, **41**, 207.

MUGHAL, A.W., HORTOBAYI, G.N., FRITSCHE, H.A., BUZDAR, A.V., YAP, H.Y. & BLUMENSCHEIN, G.R. (1983). Serial plasma CEA measurements during treatment of metastatic breast cancer. *J.A.M.A.*, **249**, 1881.

OGAWA, T., IZUO, M., MORITA, H. & 7 others (1986). Evaluation of a tumour-associated antigen CA15-3 in the sera of patients with breast cancer. *Gan No Rinsho*, **32**, 27 (*Eng. Abstr.*).

RAVRY, M., MOERTEL, C.G., SCHUTT, A.J. & GO, V.L.W. (1974). Usefulness of serial serum CEA determinations during anticancer therapy or long term follow-up of gastrointestinal carcinoma. *Cancer*, **34**, 1230.

SCHLOM, J., COLCHER, D., HORAN HAND, P. & 7 others (1985). Monoclonal antibodies reactive with breast tumour-associated antigens. *Adv. Cancer Res.*, **43**, 143.

STACKER, S.A., THOMPSON, C.H., RIGLAR, C. & McKENZIE, I.F.C. (1985*a*). A new breast carcinoma antigen defined by a monoclonal antibody. *J. Natl Cancer Inst.*, **75**, 801.

STACKER, S.A., THOMPSON, C.H., LICHTENSTEIN, M. & 4 others (1985*b*). Detection of breast cancer using the monoclonal antibody 3E1.2. In *Proc. 1st Int. Workshop on Monoclonal Antibodies and Breast Cancer*, Ceriani, R.L. (ed) p. 233. Martinus Nijhoff Pub., Boston, Mass.

STACKER, S.A., SACKS, N.P.M., THOMPSON, C.H. & 6 others (1987). A serum test for the diagnosis and monitoring of the progress of breast cancer. In *Immunological approaches to the diagnosis and therapy of breast cancer*, Ceriani, R.L. (ed) p. 217. Plenum Press, New York.

ZAR, J. (1974). Simple linear correlation. In *Biostatistical Analysis*, p. 236. Prentice-Hall Inc., Englewood Cliffs, N.J.